# Role of Faculty of Medical Colleges in National Health Policy and Program Development

**DOI:** 10.4103/0970-0218.62544

**Published:** 2010-01

**Authors:** Chandrakant S. Pandav

**Affiliations:** Centre for Community Medicine, All India Institute of Medical Sciences, New Delhi, India E-mail: cpandav@iqplusin.org

Going back into history, before the *Samudra Manthan*, even Gods suffered from illnesses and it was *Ashwani*, who treated them. There used to be a constant war between the deities and demons, but finally wisdom prevailed over them and they decided to map the treasures of knowledge, which needed to be preserved. They held a m*anthan* (churning) to extract the treasure. Traditionally, this has been associated with the churning of the *ksheer samudra, the* milk ocean. It has also been likened to a “brain storming session,” with strict rules when the merits and demerits of various alternatives are discussed. Eventually, the 14 *ratnas* emerged. Lord Dhanvantari emerged during this *manthan* and brought with him *Amrita*, the elixir of life. He is considered the God of physicians and Father of Ayurveda. Taking a cue from *samudra-manthan*, the need of the hour is to have a *vichar-manthan* or *vichar vimarsh* (churning /critically examine the ideas) to deliberate upon the role of the faculty of Medical Colleges in national health policy and program development.

I have two messages to share with you. We have all come from a culture where, if you have to make a point, you tell a story. But here, first I would like to make my points and then tell the story. I would like to recall the *Gurukul* tradition that we Indians are very proud of. In the days of the *Gurukul*, the *shishayas* (students) spent almost 12 years in the *Gurukul* to study the *Vedas,* which required extremely rigorous training. Subsequently, the Indians, being pragmatic, prepared commentaries on *Vedas* in the form of *Upanisads*. With these, the period of study reduced considerably. The term *upa-ni-sad* refers to “basically sitting down near a teacher in order to learn”.

We have a historic tradition of asking very difficult research questions in terms of the larger context of life and philosophy…. Who am I? What I should do? What is my purpose of life on this Earth? So, the first point to make is that it is extremely important for the students to spend a lot of time thinking about a research question. Spending time in identifying the research question is an investment that will always pay rich dividends.

The second message is to the faculty and concerns “mentorship”. Mentorship is an extremely challenging process. There are two kinds of teachers: those who are mentors and, those who are tormentors. It is important for the faculty to take on the challenging role of mentors. History provides us with many examples. In the mythological classic *Mahabharata*, Sri Krishna was a mentor to Arjuna, and Arjuna was a true disciple asking many questions on the entire philosophy of war, birth and human life. We have good examples of mentorship throughout the ages. Chanakya and Chandragupta Maurya of the 4^th^ century BC, personalities like Sir C.V.Raman and Homi Jehangir Bhabha in the post-independent India provide excellent examples of mentorship.

Motivation is a key factor in the relationship between student and teacher.(1) As described by David Langford, on the basis of the source of motivation, there exists a continuum: extrinsic motivation to intrinsic motivation. Extrinsic motivation basically refers to a situation wherein the students are ordered (to study). It is similar to a sincere master-slave relationship. This is followed by a “do for” situation where the teacher is the all-knowing provider and the student a passive recipient. Subsequently, in the third stage, the teacher plays the role of a coach and the student is a learning participant. Finally comes a position “enable” where the teacher is a facilitator - mentor and the student a self -learner and plays an extremely active role. As we move along this continuum, the quality of learning improves consistently with the maturing of the relationship between teacher and student, the culmination of the relationship occurs when the teacher becomes an enabler while the student becomes an active self learner.

## Values of Mentorship

There are four values of mentorship: Motivation, Inspiration, Commitment and Excellence. These are the actual routes teachers in the faculty of the Community Medicine, in the current context, need to reflect upon. As per the Regulations of Medical Council of India (MCI), thesis has been made an essential part of the degree course as this gives training in research methodology.(2) The thesis or dissertation should embody the candidate's work under a qualified supervisor for the purpose. As a faculty of the Community Medicine, it is one of our most important responsibilities to generate research questions that contribute not only to the academic progress but also play a catalytic role in formulating health policy and programs.

## How can Faculty in Medical Colleges Influence Policy?

Many of the faculties have already been involved in this activity in terms of linking research with policy and program. A simple framework that shows how medical faculty can play a role in development of policy and programs is explained by Tugwell *et al.* We will discuss this framework in detail and apply it to a case study subsequently. Briefly, the most critical step is to generate evidence through research. Research could be basic as well as applied/action research through projects, postgraduate thesis/dissertation, PhD thesis, etc. Once the research is conducted, the evidence generated needs to be disseminated mainly through scientific publications. Building goal-oriented collaborations with people who are most affected by policies and personnel who formulate the policy is an important activity as it helps translate research evidence into action or policy. Collaborations need to be established through partnerships with community, local bodies, research institutions and peers, national and international organizations. Collaborations need not wait for a study to finish. It is vital that the collaborations are involved right from planning the research. This ensures ownership of the results. But before we indulge in all of these, one must make all efforts possible to ensure that the research question asked is relevant.

## How to Generate Relevant Research Questions?

It is said that a wise man's question contains half the answer. In this context, there are five pillars of strength that the practitioners of Community Medicine can draw upon. These are (a) Experience, (b) Reading the research findings of other researchers, (c) Interactions with field workers and community and (d) Knowledge of various theories and hypothesis and (e) Awareness of type of evidence required by health programs.(3) With these inputs, the faculties will be in a position to generate relevant questions. Some of the important criteria for selection of a relevant research question are listed below.

## Criteria for Selection of a Research Question(4)

- Magnitude of the problem: whether the problem persists at international, national or regional level, the group of population it is affecting, whether it is life threatening or not
- Urgency of the need for a solution: prioritization
- Amenability of the problem to investigation
- Avoidance of duplication
- Political acceptability-interest and support of local/national authorities
- Feasibility of the approach: time expensive, labor expensive
- Chances of success
- Expected impact of a successful outcome – significant or non significant
- Expected spin-offs in terms of training of staff and other research capability strengthening elements for safe and efficient conduct of the research
- Ethical considerations
- Availability of funds or sponsorships
- Qualification and experience of the investigator.

## Research to Policy to Program: The Tamil Nadu Case Study(5)

As a member of the Indian Coalition for Control of Iodine Deficiency Disorders (ICCIDD), I was involved in a research study in Tamil Nadu trying to address the following questions:

1. The current status of Iodine Deficiency Disorders (IDD) in Tamil Nadu using the World Health Organization (WHO)/United Nations Children's Fund (UNICEF)/ICCIDD criteria
2. The availability and cost of adequately iodized salt at the household level in Tamil Nadu
3. The community perception towards IDD, salt and iodized salt in Tamil Nadu.

The IDD survey in Tamil Nadu was a collaborative project with the involvement of the government agencies (State Health Ministry, Department of Public Health and Preventive Medicine, Regional Health and Family Welfare Training Center, Food and Analysis Laboratories), State Medical Colleges, National Medical Institutes as major research centers (National Institute of Epidemiology, Indian Council of Medical Research (ICMR), Chennai, National Institute of Nutrition, ICMR, Hyderabad, All India Institute of Medical Sciences, New Delhi), non-profit non-governmental organizations (India Clinical Epidemiology Network, Micronutrient Initiative, Indian Coalition of Control of Iodine Deficiency Disorders) and international agencies (UNICEF, Tamil Nadu Branch, International Council for Control of Iodine Deficiency Disorders).

**Figure 1:**
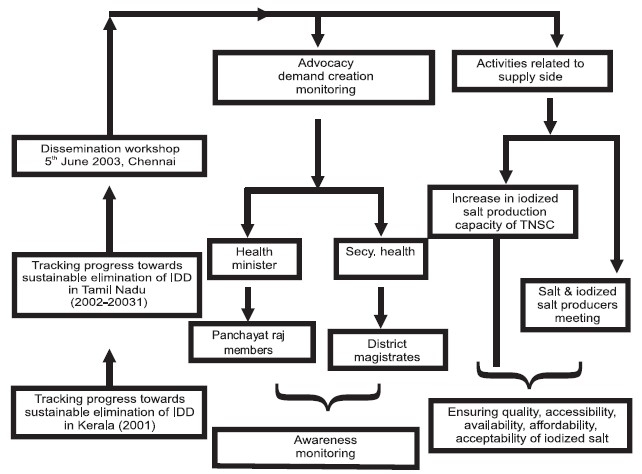
Linking research to policy & program: The Tamilnadu experience

The most important role was that of the Department of Public Health and Preventive Medicine Government of Tamil Nadu, Regional Health and Family Welfare Training Centers, Food Analysis Laboratories, and Government Medical Colleges. They undertook the important components of the project-training, field study and the laboratory analysis. This was an extremely participative, vibrant exercise where relevant research question related to the state's problem was raised and all the key actors at the state level played a key role and national and international agencies played a catalytic role contributing to this exercise. IndiaCLEN helped with the qualitative study related to the last two questions. The overall coordination and provision of technical expertise was done by ICCIDD. For funding, the agencies responsible were Micronutrient Initiative, New Delhi and UNICEF, Tamil Nadu Branch. WHO/ICCIDD/UNICEF criteria for tracking progress towards eliminating IDD as a public health problem were followed. The following were the main findings: goiter prevalence in 6-12 year olds 13.5% (goal <5%), median urinary iodine 89.5 μg/L (goal >100μg/L), and proportion of households consuming adequately iodized salt 18.2% (goal >90%). These findings indicated surprisingly poor iodine nutrition in a state that is among the leading states in India in many health indicators [Figure 1].

However, that was not the end and the study was followed up by a dissemination workshop in June, 2003, organized by the Government of Tamil Nadu. Participants included Government of Tamil Nadu and its agencies, Tamil Nadu Salt Corporation, National Institute of Epidemiology, ICMR, Chennai, National Institute of Nutrition, ICMR, Hyderabad, All India Institute of Medical Sciences, New Delhi, IndiaCLEN, The Micronutrient Initiative, New Delhi, ICCIDD, UNICEF, Tamil Nadu Branch and International Council for Control of Iodine Deficiency Disorders. The recommendations of the Workshop were to establish an IDD Review Committee at the Department of Public Health and Preventive Medicine with due representations from various stakeholders, to promote awareness in the community through television and radio, grassroots level workers through the health system, to strengthen production level monitoring and establish a system of annual cyclic monitoring.

The Health Minister and the Health Secretary wrote to the Panchayat Members and District Magistrates to focus on monitoring and awareness generation. From the supply side there was a move towards increased iodized salt production where the Tamil Nadu Salt Corporation played an important role. There were a series of meetings with the salt producers regarding the same. The main objectives were to ensure quality, accessibility, availability, and affordability of iodized salt.

With these activities, we could link research to policy and program. Some of the immediate outcomes were (1) Quality iodized crystal salt distributed at Rs.2.50/kg through the state Public Distribution System (PDS), (2) Increase in distribution of iodized salt by ten folds through PDS from 4,200 tons in year 2000 to 42,829 tons in the year 2004, and (3) Distribution of salt to the below poverty line population through PDS.

## Conclusion

I would like to conclude by sharing with you the following eight principles, which will enable Medical Faculty in influencing policy and program;

1. Postulating well-defined research questions
2. Sound scientific research protocol with qualitative and quantitative components
3. State specific information
4. Inter-disciplinary contribution
5. Participatory approach
6. Partnership with stakeholders
7. Linking research to policy to program
8. Tracking progress– monitoring and evaluation

In terms of making a real difference to policy, the researchers/faculty members of medical colleges need to be much more proactive. A more critical aspect is establishing partnership of stake holders. Finally, in developing programs, the value of evidence for informed decision making is of great importance and this transition from evidence to programs is linked with forging partnerships with stakeholders. In terms of advances with the launch of the National Rural Health Mission (NRHM), people and civil society are becoming important tools for external monitoring of programs. And again, here, the medical faculty has a great opportunity in influencing policy by answering questions related to program effectiveness.

In conclusion, I would also like to share with you another important story which is relevant to today's oration of post independent era. I share with you a classical example of mentorship of Sir C.V. Raman, the Nobel Laureate in Physics for his discovery of Raman Effect. Dr. Raman was the in the first batch of Bharat Ratna awardees. The award ceremony was to take place in the last week of January, 1954, soon after the Republic Day celebrations. The then President, Dr. Rajendra Prasad, wrote to Dr. Raman, inviting him to be his personal guest at Rashtrapati Bhavan during that time. Dr. Raman replied politely, regretting his inability to come to Delhi and attend the ceremony. Dr. Raman had a noble reason. He explained to the President that he was guiding a Ph.D. student whose thesis was due by the last day of January. The student was valiantly trying to wrap it all up, and Dr. Raman felt he had to be by his side and see that the thesis was finished, sign it as the guide and then have it submitted. Here was the scientist who gave up the pomp of a glittering ceremony associated with the highest honor because he felt his duty required him to be by the side of the student. It is this character that truly builds science. This is one of the experiences how leadership can contribute to the growth of science. One of the greatest gifts we can give to another generation is our experience and wisdom. I would like to end with words by Robert L. Frost, “*Two roads diverged in a wood, and I – And I took the one less traveled by- And that has made all the difference”.*
